# Efficacy of breast cancer screening program in Kingdom of Saudi Arabia

**DOI:** 10.15537/smj.2022.43.4.20210823

**Published:** 2022-04

**Authors:** Khalid A. AlSaleh

**Affiliations:** *From the Department of Medical Oncology, College of Medicine, King Saud University, Riyadh, Kingdom of Saudi Arabia.*

**Keywords:** breast, screening, cancer

**B**reast cancer (BC) is a globally challenging health concern and has become the most common type of cancer among women. The disease is associated with a significant health burden and mortality among women. In 2020, 2.3 million women were diagnosed with BC, and 685,000 deaths were reported globally. Some 7.8 million women had been living with BC for the past 5 years, making it the world’s most prevalent form of cancer.^1^

The Kingdom of Saudi Arabia (KSA) comprises over four-fifths of the Arabian Peninsula with 30 million inhabitants. According to an incidence report published by the Saudi Cancer Registry in 2017, BC ranked top among women and contributed to 18.1% of all cancers.^2^ Breast cancer accounts for 30.9% of all cancers cases reported among women of all ages, with median age of 51 years (range= 20-117 years) at the time of diagnosis.^2^ In 2010, BC was the ninth leading cause of death among women in KSA. The incidence of BC is expected to rise in the coming years in KSA due to population growth and aging. Timely diagnosis of at-risk or affected patients can help reduce the BC-associated mortality. Despite the proven effectiveness of BC screening in reducing mortality, low uptake rates have been reported in Arab women. Three main screening methods are used in KSA: self-breast examination, clinical breast examination (CBE), and mammographic screening. The Center for Disease Control and Prevention (CDC) recommends mammography for screening BC every two years for women aged 50-74 years.^3^

Mammographic screening is considered the gold standard for BC screening, reducing mortality by 23%.^4^ Mammography was introduced in KSA in 2002. Saudi Arabia’s Ministry of Health provides free mammography screening services. A nationwide BC screening center was inaugurated in 2007, with 1,215 individuals screened for BC in the first year of its operation.

Additionally, a regional mammography screening program was organized in 2007 in Al Qasim that targeted women aged 35-60 years and was also preceded by an awareness campaign. In addition to these mass screening centers and programs, mammography has been available in all the regions of KSA since 2005. Despite the countrywide availability of screening services, the National Saudi Health Interview Survey 2015 reported a significantly lower breast cancer screening rate (8%). This is considerably lower than the screening rates in the United Kingdom (72%; 2019-2020),^5^ the United States (76.4%; 2019),^6^ France (49.9%; 2017),^7^ and Sweden (75-85%).^8,9^

Many barriers to BC screening have been identified globally, including access to health services, difficulties within infrastructure, incomplete information, ethnicity, socioeconomic status, and geographical conditions. In KSA, a significant number of women presented with advanced stages of BC owing to the lack of information, knowledge, and awareness of screening and early detection measures. Although knowledge gaps regarding BC and associated risk factors among female high school students have been reported, awareness is higher among the older female population. The screening rates remain lower for women in KSA, with 57% reporting performing a self-exam, while 89% of the respondents reported not having a CBE in the past year and 92% reported never having a mammogram.

Furthermore, the screening rate varies widely across different geographical regions of KSA. Few studies examined the possible predictors for the late presentation of BC among local women despite free screening programs and services. Exploratory factor analysis has identified personal fears related to the healthcare system; examination and results contribute to 30.4% of the barriers.^10^ Well-planned and proactive screening programs utilizing systematic calls, recalls, follow-ups and surveillance are more efficient for early detection. Organized cancer screening programs reduce cancer mortality and are more cost-effective than opportunistic programs are. The yield of a structured mammogram screening program in Saudi Arabia was high, indicating that a more effective screening program can detect early stage BC. Coronavirus disease (COVID-19) has created a new opportunity to utilize technology to contain the spread, and the KSA was a pioneer in this regard through the digital transformation of its healthcare delivery systems.^11^ The same strategy can be applied to detect the early stages of BC through screening, especially in the high-risk population. We suggest transforming the current opportunistic BC screening programs in KSA into organized programs that can enhance screening rates and reduce the BC disease burden.

The age-standardized incidence of BC has increased over the years in KSA.^12,13^ This could be attributed to an increase in the average life expectancy in KSA, with a concomitant increase in the high-risk population of older women.^12-15^ In developing countries, the incidence of BC is rising and becoming similar to that in developed countries.^16^ The lower incidence rate of early BC in KSA compared to the developed countries may be partly due to the reduced sensitivity and coverage of screening programs.^2^ This emphasizes the need for more intensive screening programs across KSA.

The BS stage at diagnosis is a predictor of resource utilization, with patients at advanced stages requiring significantly costly interventions compared to their early stage counterparts. The total yearly cost of treating BC in KSA was estimated to be $13.3 million. While the total cost of treatment for stage I patients was estimated to be around $569,953, it increased exponentially to $7,822,911 for stage IV patients. The average yearly cost per stage IV per patient was $81,489 in KSA compared with $62,108 in the United States.^17^ Breast cancer detection at an early stage not only drives down the cost significantly lowering the health burden, but it also reduces mortality. This highlights the need for an effective screening program with extensive and uniform coverage. Therefore, the screening programs must be included in routine healthcare and diagnostic services, where the high-risk populations must be screened regularly on a priority basis to ensure an early diagnosis.

There is a growing need to build a new screening model integrating private and public hospitals emulating the prevalent COVID-19 screening across KSA, as the previous program does not meet its target coverage for early detection to reduce the disease burden. Additionally, regular awareness programs must be carried out to empower the populace with knowledge of risk factors, health dangers, and the efficiency of screening programs for early detection and effective management of BC. Lack of proper training has been identified as a significant factor responsible for the low rates of self-exam among Saudi females. Such awareness programs must also include self-exam training sessions.

In summary, BC is the most prevalent form of cancer. Early detection can help improve disease prognosis. In KSA, self-examination, CBE, and mammography are used for BC screening. However, screening rates are not optimal despite free and widespread screening services. The cost of screening programs is much lower than the cost of treatment, especially in the later stages of the disease. Thus, an extensive update of the current screening policy is required to introduce mass screening programs that facilitate early BC detection and lower mortality. Awareness campaigns may help improve screening rates and reduce BC-associated mortality among women.
